# Disaster victim identification operations with fragmented, burnt, or commingled remains: experience-based recommendations

**DOI:** 10.1080/20961790.2020.1751385

**Published:** 2020-05-26

**Authors:** Hans H. de Boer, Julie Roberts, Tania Delabarde, Amy Z. Mundorff, Soren Blau

**Affiliations:** a Department of Forensic Medicine, Netherlands Forensic Institute, The Hague, The Netherlands; b Department of Pathology, Amsterdam UMC, University of Amsterdam, The Netherlands; c Faculty of Science, School of Biological and Environmental Sciences, Liverpool John Moores University, Liverpool, UK; d Principal Forensic Services Ltd, Bromley, UK; e Institut Médico-Légal de Paris, Paris, France; f Université de Paris, BABEL, CNRS, Paris, France; g Department of Anthropology, University of Tennessee, Knoxville, TN, USA; h Department of Forensic Services, Victorian Institute of Forensic Medicine, Melbourne, Australia; i Department of Forensic Medicine, Monash University, Melbourne, Australia

**Keywords:** Forensic sciences, forensic anthropology, DVI, fragmented human remains, burnt, commingled, DNA

## Abstract

Human-made and natural disasters can result in severely fragmented, compromised, and commingled human remains. The related disaster victim identification (DVI) operations are invariably challenging, with the state of the remains potentially precluding some identifications. Practitioners involved in these DVI operations will routinely face logistical, practical, and ethical challenges. This review provides information and guidance derived from first-hand experiences to individuals tasked with managing DVI operations with fragmented human remains. We outline several key issues that should be addressed during disaster preparedness planning and at the outset of an operation, when incident-specific strategies are developed. Specific challenges during recovery and examination of fragmented remains are addressed, highlighting the importance of experienced specialists at the scene and in the mortuary. DNA sample selection and sampling techniques are reviewed, as well as downstream effects of commingling and contamination, which can complicate reconciliation and emphasise the need for rigorous quality control. We also touch on issues that may arise during communication with families. While recommendations are provided, they are not intended as proscriptive policy but rather as an addition to the general recommendations given in the International Criminal Police Organization (INTERPOL) DVI Guide, to inform preparative discussions between government officials, judiciary, police, and forensic specialists.

**Key points**
- A DVI operation for an incident characterised by many fragmented and otherwise compromised human remains poses specific challenges that may prolong and complicate identifications.
- Specialists should be consulted at the outset to address key issues related to the aim and extent of the operation.
- Specialist expertise in handling compromised human remains is indispensable at the scene, in the mortuary, during reconciliation, and for quality control.
- Continuous consultation between representatives from government, the judiciary, law enforcement, the media, and various forensic specialists will prevent unnecessary delay and facilitate accurate and timely communication.

## Introduction

Disasters, either natural or human-made, are inherently chaotic events with major adverse effects on infrastructure, environment, and communities. When a disaster involves large-scale loss of life, the recovery process is further complicated by a simultaneous identification operation. The primary aim of a disaster victim identification (DVI) operation is to recover all human remains, identify the deceased, and certify their cause and manner of death. Additionally, the investigations related to DVI may be instrumental in reconstructing the cause of the event, which can facilitate the development of preventive measures.

In 1984, the international police network International Criminal Police Organization (INTERPOL) introduced the first written DVI guidelines. The INTERPOL DVI Guide is published and regularly updated by a DVI working group advised by four scientific sub-working groups related to the areas of forensic expertise generally consulted in DVI operations: odontology, pathology/anthropology, ridgeology (fingerprints), and molecular biology (DNA). DVI operations worldwide typically follow the INTERPOL DVI Guide and use its accompanying documentation forms [1]. The INTERPOL DVI Guide provides a framework to manage mass fatality incidents but does not specifically address issues related to events with many fragmented human remains [2]. The documentation forms have proven effective in disasters with relatively intact bodies, but may not be appropriate when used for complex DVI operations with fragmentary and compromised remains.

Incidents with severely fragmented, compromised, or commingled human remains pose complex logistical, practical, and ethical challenges that may prolong the DVI operation or even preclude identification of some individuals altogether. Maximising identification efforts in such circumstances requires a different approach to documentation in the mortuary, as well as detailed planning and effective communication between government officials and forensic practitioners. The importance of an augmented approach to complex incidents is even greater with the increased sensitivity of DNA techniques and more sophisticated ways to interpret mixed or partial DNA profiles. These developments have made it possible to identify even the smallest piece of human tissue [3,4], but they also pose procedural and ethical questions for incidents characterised by fragmented and compromised remains.

Furthermore, there are limited experience-based resources for addressing the abovementioned challenges. This article therefore presents an overview of lessons learned from several complex DVI operations, all characterised by severely fragmented and compromised human remains. First, complex operations in which the authors have been involved are summarised, followed by a discussion of key issues in DVI preparedness planning. Remains recovery and examination, DNA sampling, reconciliation, and quality control are reviewed, as is a consideration of issues that may arise during communication with community groups and next of kin.

## Materials and methods

The recommendations listed in this publication are based on the first-hand experiences of the authors, all of whom are experienced forensic anthropologists with one (HdB) also being a forensic pathologist. The authors have worked on numerous DVI operations with many fragmented remains; a summary of the major incidents that form the basis of the recommendations is provided below.

### September 11 terrorist attack on the World Trade Center

On 11 September 2001, nine terrorists flew passenger aircraft American Airlines 11 and United Airlines 175 into the north and south towers of the World Trade Center in New York City. Within 2 h, both towers collapsed, killing nearly all civilians, first responders, and emergency personnel inside. The disaster site covered 64 500 m^2^, was 43 m deep, and had three “hot spots” burning for almost 3 months. The fires were periodically doused with brackish water from the nearby East River. It took nearly 9 months to comb the debris field and recover approximately 22 000 fragments of victim remains. These human remains were extremely fragmentary, decomposed, burnt, and commingled. More than 3 years were required to finalise the missing persons list. As of August 2019, 14 696 human fragments have been identified and matched to 1 637 of the 2 749 victims. Primary mortuary operations lasted approximately 11 months and were undertaken by a multidisciplinary team of medical examiners, an anthropologist, New York Police Department fingerprint examiners, forensic odontologists, X-ray technicians, medicolegal investigators, DNA analysts, and hundreds of volunteers. Quality control checks and reanalysis lasted an additional 6 months after initial analyses were complete in the mortuary, and DNA testing and resampling are still ongoing.

### American Airlines 587

On 12 November 2001, passenger aircraft American Airlines 587 crashed in a residential neighbourhood in Queens, New York less than 2 min after take-off. All 260 people on board, along with five individuals on the ground, were killed. The National Transportation Safety Board determined the cause of the crash to be a combination of an airline design flaw and pilot error [5]. Over the course of a few days, 2 100 fragments of human remains were recovered from the ground surface, and 305 were intact enough to warrant autopsy [4]. The remains of multiple individuals exhibited thermal injuries from fuel-induced fires, but little calcination or commingling occurred. The human remains were sent to the Office of the Chief Medical Examiner in New York City, the same office working on the World Trade Center identifications, and the same DVI process was used. Autopsies were completed in 7 d, and nearly 1 800 fragments were processed in 12 d. All victims were identified within 28 d [6].

### The “Black Saturday” bushfires

On 7 February 2009, the state of Victoria, Australia experienced extreme heat and strong winds. In combination with faulty overhead power lines, arsonists, and lightning strikes, this heat event caused a total of over 300 bushfires in 4 500 km^2^ that resulted in the deaths of 173 people. Total extinguishment of the fires took approximately 6 weeks [7]. Recovery of the highly fragmented and predominantly burnt remains took several weeks and included numerous revisits to disaster sites [8]. Very few intact bodies were recovered, and multiple bodies were often found at one location. After communication with the coroner, all remains were examined at the Victoria Institute of Forensic Medicine by forensic pathologists, substantially assisted by a radiologist, forensic anthropologists, odontologists, and (in selected cases) fingerprint specialists. Thousands of bone fragments were examined but owing to the scale of the event, the exact number of fragments was not recorded. Final identifications were formally made 3 months after “Black Saturday”.

### The Malaysia Airlines 17 crash

On 17 July 2014, passenger flight Malaysia Airlines 17 from Amsterdam to Kuala Lumpur was shot down while flying over Ukraine, killing all 283 passengers and 15 crew members on board [9]. An armed conflict at the disaster site delayed the recovery operation and necessitated the shipment of the remains to Hilversum, the Netherlands, where an international DVI operation was led by a Dutch DVI team. The multinational DVI team included fingerprint specialists, forensic pathologists, anthropologists, radiologists, odontologist, and DNA experts. Most of the recovered remains were heavily fragmented, decomposed, and/or thermally altered, which necessitated numerous re-examinations because of commingling and cross-contamination. In the spring of 2015, thousands of small, mostly skeletonised human remains were also recovered and examined, which increased the number of DNA samples to more than 8 000 from 296 of the 298 victims [2,10,11]. As of October 2019, the DVI operation is officially still ongoing, with sporadic findings of human remains from the 30-km^2^ crash site submitted for examination.

### The Shoreham airshow crash

On 22 August 2015, 11 people were killed when a vintage Hawker Hunter jet crashed onto a busy dual carriageway during an airshow in West Sussex, UK. The impact from the jet caused major bodily disruption, with dispersal and commingling of body parts across a wide area. The search and recovery of the remains took approximately 3 weeks, with further damage and decomposition of human remains caused by fluctuating weather conditions. Despite the relatively small number of victims, more than 1 200 body parts were recovered and the examinations lasted approximately 6 weeks, with numerous re-examinations required because of commingling and potential cross-contamination. All individuals were identified. A forensic anthropologist and archaeologist assisted at the scene. A forensic pathologist led the mortuary examinations during the first 5 d, focussing on the cause of death and larger body parts. An anthropologist assumed responsibility for the subsequent 5 weeks of examinations, assisted by anatomical pathology technicians and a police DVI mortuary team.

### November 2015 Paris attacks

On the evening of 13 November 2015, three groups of men launched six separate assaults—suicide bombings and mass shootings—in the centre of Paris and the northern suburb of Saint-Denis. In total, 130 people were killed [12]. Many intact bodies were recovered along with 129 fragmentary and commingled body parts. Partially owing to inexperienced recovery technicians, numerous re-examinations in the mortuary and revisits to the scenes were necessary [13]. A multidisciplinary team of specialists that included forensic pathologists, odontologists, radiologists, fingerprint police specialists, and one forensic anthropologist examined all bodies and body parts. All individuals were identified in 1 week, mostly using DNA. Pressure from judicial authorities and investigators to identify the perpetrators as soon as possible resulted in the successful separation of the perpetrators from the victims. Five days after the attack, two escaped perpetrators were arrested, at which time they detonated a bomb vest, killing themselves and an accomplice [14]. The approximately 90 body parts of these individuals were analysed by the DVI team as a separate event, and their identification took approximately 6 months.

### Rue Erlanger apartment block fire

On 5 February 2019, around midnight, a fire in a Paris apartment block killed 10 people and injured an additional 40. The fire was alleged to have been started intentionally. The fire was extremely intense, quickly consuming several floors. Eighty-five fragmented and charred bodies/body parts were recovered under supervision of a forensic anthropologist and odontologist. The charred bodies and body parts were first CT-scanned, after which radiologists and the anthropologist triaged the remains. The remains were subsequently examined by a multidisciplinary team of forensic pathologists, odontologists, and a forensic anthropologist in close collaboration with a DVI police unit. All individuals were identified over 4 days. Most of the victims were identified using DNA.

## Lessons learned

Each disaster is unique, and the final decision on the mandate, extent, and procedure of each DVI operation is the prerogative of the local legal authorities. As such, the lessons learned set out below are not intended as proscriptive policy. Rather, they are meant to guide preparative discussions between relevant government officials, judiciary, police, and forensic specialists. The discussion is not meant to supplant the INTERPOL DVI Guide, but to provide additional information specifically for incidents with many fragmented remains.

### Early decision-making and communication by policy makers and judiciary officials

All abovementioned incidents have shown that a dedicated strategy to managing human remains will help ensure relative order in the complex situation following mass fatality. Ideally, this strategy should be part of the disaster preparedness plan rather than reactionary. Because each disaster carries its own intricacies, such a plan should not be restrictive and should include an outline of key topics that should be addressed at the outset of a DVI process. Ideally, these topics should include the following considerations:

- Who will advise the responsible decision-makers? In the moments following an incident, obtaining reliable information is key to the timeliness of remains recovery and establishing identifications. The importance of providing experienced and specialist advice to the local authorities at the scene of the disaster cannot be overstated, especially when dealing with compromised bodies.
- Who will be responsible for the DVI operation, and what is the chain of command? Typically, local authorities will have addressed this in their response plan. However, confusion about the role of forensic specialists may still cause considerable delay, particularly if there are multiple scenes.
- Which experts are required at the scene? Relevant expertise at the scene, especially forensic archaeologists or anthropologists when human remains are fragmented or burnt, has proven to be essential for efficient recovery. The scale of the 2009 Victorian bushfire incident, coupled with multiple concurrent scenes and limited resources, highlights the need for expertise at the scene. Many of the initial scene attendances did not include a forensic anthropologist or archaeologist, experienced with recovering small burnt and fragmentary bones, and therefore required numerous site revisits. The same was true following the World Trade Center recovery operation, which was predominantly undertaken by the New York Fire Department. Their inexperience resulted in avoidable and additional commingling at the site, which then had to be addressed during the mortuary process. The number of deceased is not the single most important factor when selecting specialists; at the Rue Erlanger fire incident in Paris, an anthropologist and odontologist attended the scene for (only) 10 fatalities to aid in recovery and prevent additional commingling.
- Forensic anthropologists routinely attend fatal fires in the UK to record the location of victims and disassociated fragments, which can be complex. For example, in 2017, six family members were killed in a fire in Wales. The victims had fallen through several floors and some were commingled, giving the impression that they had been together at the time of death. Careful excavation and examination of the remains by forensic anthropologists and archaeologists established that this was not the case and that they had, in fact, been in separate rooms in different parts of the house. Scene analysis contributed to the interpretation of what had occurred on the night of the fire, and proved crucial to the coronial and police investigations. These examples highlight the fact that even when the number of victims is relatively low, specialist assistance at the scene can significantly contribute to a successful DVI operation, particularly if the remains are extremely fragmented and dispersed or exhibit significant thermal alterations and commingled.
- To what extent will postmortem examinations be undertaken? This decision is often context-specific. When the cause of death is assumed, or there is no suspicion of criminal intent, local authorities may decide that the examination of the remains will be limited to identification. This can minimise the mortuary phase of the DVI operation considerably. For example, cause and manner of death from the World Trade Center disaster were predominantly homicide/blunt force trauma, and autopsies were not conducted. However, during the DVI operation for the victims of American Airlines 587, autopsies were legally mandated by federal laws. During the Malaysian Airlines 17 operation, autopsies were limited to specific persons of interest, such as the pilots. Close communication with the local authorities is vital. Following the 2009 Victorian bushfires, any suspicion of a death other than by fire was communicated to the coroner and the extent of the postmortem examination was reviewed [15].
- What is the identification mandate? Will every fragment be identified, if possible, or will the sole goal be victim identification? In disasters with a closed population and a known missing persons list, authorities may decide to end the DVI operation as soon as each individual is accounted for. In an open-population disaster, an unknown number of victims precludes such an approach. Discussion should then focus on the level of identification, i.e., what type or size of human remains is considered “identifiable”. Often, this decision is dictated by resource availability.
- Terminology must be clear and specific. Non-specific terminology may cause confusion or even lead to erroneous conclusions. For example, terms such as “full body”, “body part”, and “fragment” should be clearly defined. Specific descriptions are also needed to clarify context when interdisciplinary personnel are working together on a DVI operation. For example, the debriefing following the 15 March 2019 mass shootings at mosques in Christchurch, New Zealand revealed that to DVI forensic medical experts, the term “fragmentation” indicated body preservation, whereas for police investigators, the term related to ballistic evidence (Supt. P. Jermy, pers. Communication, 2019).
- How will information be conveyed to families of the victims, the broader community, and government officials? Families may have specific questions regarding potential survival time and completeness of the body. Managing expectations and communication with next of kin are imperative at the outset, especially if it is possible that a number of fragments would not be identified. Additionally, special attention should be paid to how body fragmentation may impact specific religious and cultural preferences. For example, some religions consider it essential to recover all body parts for burial. Realistic expectations should be communicated to all relevant parties simultaneously and consistently.
- Is there a media communication strategy? Well-informed and timely media briefings ensure that misinformation is not disseminated. A dedicated spokesperson will contribute to uniformity in the shared information.

Typically, the complexity and duration of a DVI operation with many fragmented remains are underestimated. Discussion of the abovementioned topics with all stakeholders can mitigate this to some extent. The need for rigorous, quality-checked identification methods should always prevail over speedy identifications.

### The scene

Incorrect recovery techniques at the scene adversely affect mortuary operations, as demonstrated in some of the DVI operations above. Examples include grouping multiple sets of fragmentary remains in the same recovery bag and attempted body reconstruction at the scene [3]. To circumvent some of these problems, a specialist in identifying and collecting differentially preserved human remains should be included in the initial strategy meeting immediately following the incident. During the initial site assessment, such a specialist can help evaluate the condition of the remains, assist in formulating the strategy for recovery, and collect data *in situ*.

No two disaster sites are identical; therefore there is no single approach suitable for every context. For example, an unmanned aerial vehicle survey and laser scanning might be appropriate to map a large open area such as a plane crash site, where remains are scattered across the ground surface [16,17]. However, where a large amount of debris is present, remains are buried and excavation is required, such as a building explosion or fatal fire, a total-station surveying technique and photogrammetry on the ground may be more appropriate. Buried and fragmentary remains may require establishing a grid over the site, to record recovery location either manually or by GPS. Accurately recording distribution patterns at the scene is vital for accident reconstruction or criminal investigations, coroners’ inquests, and public and private inquiries. These types of disasters may also necessitate large-scale sieving programmes, which require input from specialists in recognising human remains.

Special consideration should be given when recovering burnt remains, as they are often highly fragmented and extremely fragile. They may be dispersed over a wide area, commingled with the remains of other individuals, or mixed in with other non-human material. Intense heat can alter the size, colour, shape, and mechanical properties of human tissue, making the recognition of burnt remains difficult [18–21]. If bone is burnt to the point of calcination (white, brittle, and with no organic material surviving), obtaining a DNA profile is impossible [22]. Therefore, accurately recording the positions of burnt fragments at the scene is imperative for re-association if DNA identification is not possible. Reliable associations can be identified *in situ*, which allows re-assignations with larger, identifiable body parts in the mortuary. This approach was employed by a forensic anthropologist while excavating a tank that had been hit by an improvised explosive device in Afghanistan in 2012, killing all six soldiers on board. The large amount of ordnance that they had been carrying intensified the explosion and prolonged the burning inside the vehicle. Prior to excavation and recovery of the fragments, a plan of the vehicle’s interior was made, dividing it into zones corresponding with the original locations of the soldiers. This assisted greatly with the re-assignation of body parts and the preliminary identifications in Afghanistan. Formal identifications were completed in the UK following joint examinations with a forensic pathologist.

**Figure 1.**
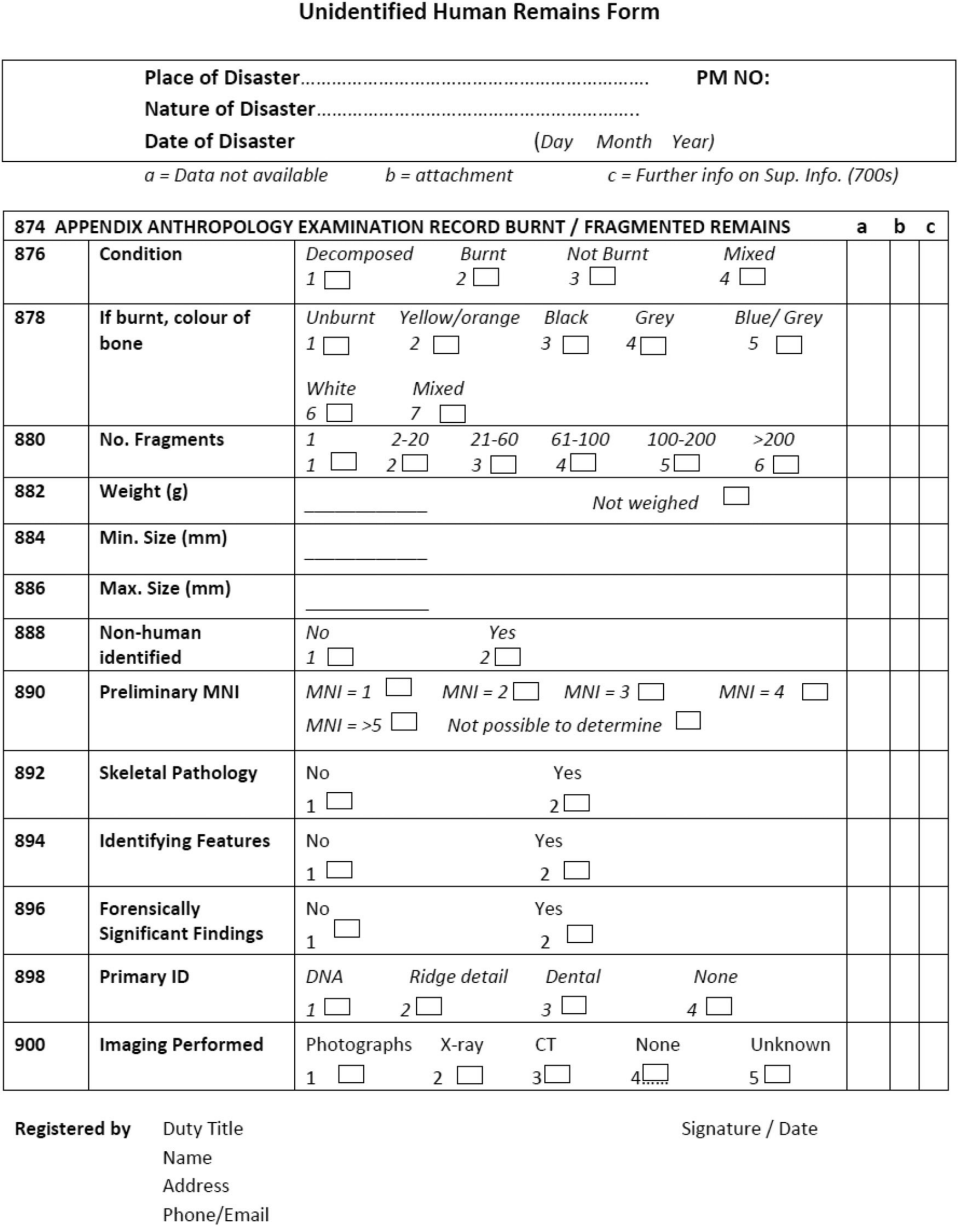
Unidentified fragmentary remains form. Note this fragmentary remains form can be used at various stages during a disaster victim identification (DVI) operation. At the disaster site, the form can be used to document single or multiple fragments, providing a rapid reference for transportation and further examination. At the mortuary, the form can be used to document triage or during detailed examination of single fragments. Because the form can be used at various phases of the operation and can relate to single or multiple fragments, a rigorous numbering strategy should be used. This form is currently considered for inclusion in the International Criminal Police Organization (INTERPOL) DVI Guide, and follows the general layout of INTERPOL DVI forms. MNI: minimum number of individuals.

Quality assurance should be integral to the recovery process. If the recovery is not undertaken by a specialist with expertise in compromised remains, the victim recovery forms accompanying the body parts should be reviewed before the body bags are sealed. This ensures that the descriptions of remains are correct and that accurate sketch plans of locations have been completed. A new form for recording and packaging highly fragmented human remains has recently been designed (Figure 1) and is currently under consideration by the INTERPOL DVI working group. The form can be used for multiple purposes, one being the labelling of single or multiple fragments during recovery. When filled in at the recovery site, the form can provide a rapid overview of key information that will guide further processing of the recovered materials.

Forensic anthropologists are often the specialists that assist with compromised human remains [6, 23–26]. As such, forensic anthropology is now listed by INTERPOL as a key discipline in DVI operations with many fragmentary and burnt remains [27]. When the deceased are in various states of completeness, a forensic anthropologist can be responsible for recording, mapping, and recovering the burnt and fragmented remains, while police DVI teams recover whole bodies and large body parts.

### Triage

Full bodies and larger body parts are typically prioritised at the beginning of a DVI operation, with fragments addressed later in the process. Forensic practitioners, however, have an ethical responsibility to examine all human remains regardless of size or preservation status and despite their specific challenges [15]. Small fragments may have identifiable features, such as ridge detail, which can assist or progress positive identification. In situations where the recovery has not been performed by a specialist, initial examination as part of a triage process can help to eliminate obvious non-human material and prioritise the human remains for examination (that is, usefulness for identification) and collection of DNA samples. Because the standard INTERPOL forms are too elaborate for fragmented remains, the “fragmented remains form” (Figure 1) can also be used during the examination. During triage, fragments will regularly be singled out from a group of recovered fragments, that is, as a result of unnoticed commingling. This requires a rigorous documentation and numbering strategy. The initial examination should be undertaken by a forensic pathologist and a forensic anthropologist. A combination of imaging and physical inspection has proven to be of considerable value [28–30]. Ideally, the contents of the body bag should also be photographed.

### The mortuary

While the initial examination involves triage, a more detailed examination, conducted as part of the mortuary assembly line, is geared toward identification. There is significant value in this examination being undertaken by a forensic anthropologist while a forensic pathologist attends to the larger, more readily identifiable bodies or body parts. Simultaneous examination ensures that the fragments are sampled for DNA as soon as possible, instead of waiting until the larger parts have been processed. This not only limits further decomposition and degradation but also allows for identified bodies or body parts to serve as DNA exemplars for unidentifiable fragments without identifying characteristics. At the same time, in instances with closed populations and limited resources, the possibility of 100% victim identification may be achieved before 100% fragment identification.

A stepwise approach is best for the detailed examination of fragments, initially removing non-human material inadvertently collected at the scene and then separating duplicate skeletal elements, an indicator of commingling. Re-association of fragments may reduce the number of DNA samples needed. This should, however, be limited to actual physical articulation of fractured bones, and the slightest suspicion of commingling should prompt separation of fragments. As fragmented remains may be separated and re-associated with other fragments several times during the DVI operation, a rigorous documentation strategy that includes photographs and chain of custody is required. This will prove extremely valuable should future re-examination and re-sampling become necessary.

A detailed examination of each fragment should always be performed, initially to determine the presence of a primary identifier outside DNA, i.e., fingerprints or odontological information. Additionally, it is necessary to record specific features that may assist with positive identification (such as skeletal anomalies or surgical intervention) or the reconstruction of the events (such as relevant forensic findings or patterns of perimortem trauma) [31–33]. However, even when such features are not present, a detailed description of each fragment can be essential for identifying commingling, cross-contamination, laboratory mistakes, and bag or sample mis-numbering [6]. Again, the form in Figure 1 can be used to document the examination of fragments instead of using the standard INTERPOL forms, which are not designed for documenting small fragments.

Incidents with prolonged recovery operations and thousands of fragments may require weeks, months, or even years to complete the analysis of all remains. During this period, the remains should be stored in such a way that minimises further decomposition. One option is to keep the remains frozen, preferably at −80 °C, although the necessary infrastructure may be prohibitive for larger fragments. An alternative approach is to desiccate the remains prior to storage. DNA testing following desiccation of remains from the World Trade Center disaster indicates that this method prevents further decomposition without negatively impacting genomic information.

### DNA sampling

Numerous studies [22, 34–40] have investigated types of human tissues for sampling, with sample recommendations ranging from buccal swabs to blood, cartilage, and bone. However, unless samples are taken from intact bodies, soft tissue has a higher risk of cross-contaminated DNA because it is more difficult to clean than a bone sample [3]. Therefore, sampling soft tissue from small, fragmentary, and potentially commingled remains is not recommended.

Various studies [34,35,38,41] have examined the skeletal elements that yield the highest quality and quantity of DNA. Some bones have higher DNA reservoirs, with cancellous bone tissue outperforming cortical bone tissue. This benefits testing when remains have been contaminated or cannot immediately be tested. Advances in laboratory procedures, however, mean that most skeletal elements yield full profiles [41,42]. Our experience shows this to be true, which renders element selection obsolete in recent DVI operations. In the Shoreham air crash, for instance, a 100% success rate for identification using bone was achieved, irrespective of the element analysed. The results of the Malaysia Airlines 17 DVI operation [2] also show an unexpectedly high success rate, even for the minute fragments submitted during the later phases of the operation, when every unburnt bone fragment weighing more than 4 g was submitted for DNA analysis.

Bone outperformed tooth samples in the Malaysia Airlines 17 investigation [2]. This finding was not unexpected as teeth are a good DNA reservoir, but their processing often necessitates boutique procedures that are unrealistic for large-scale DVI operations. If a tooth is to be submitted as a DNA sample, it is vital that it is first subject to odontological analysis.

During the DVI operation, close liaison between the mortuary and the DNA laboratory increases operational efficiency [43]. DNA specialists can provide feedback on which samples have a low yield, or which type of samples generates multiple profiles.

With fewer practical limitations on DNA testing, the decision whether to test every fragment becomes an important consideration early in the operation. Authorities should be fully cognizant of the effects of a decision to do “everything possible”. Not only can this lead to potentially unrealistic expectations (for example, retesting of the remains recovered from the World Trade Center is still ongoing), but it will also increase the cost and duration of the DVI operation considerably. Moreover, it will significantly impact the regional availability of DNA analysis outside the DVI operation.

Sampling DNA in a DVI mortuary does not differ much from sampling DNA during normal autopsy practice. However, special attention should be paid to limit cross-contamination. In soft tissue samples, this means that after any incision through an outer layer, the scalpel should be discarded (if disposable) or decontaminated with 10% bleach prior to obtaining an uncontaminated sample from the inner tissue. Bone can be sampled using a disposable scalpel if it is an intact specimen such as patella or foot bone. Sampling intact bones can also prevent additional contamination during bone sectioning. Additionally, bone can be sampled by hand-sawing a section using a junior hacksaw or autopsy saw, although this may require electricity and can be labour-intensive, particularly if practitioners are sampling hundreds of cases per day. Unless disposable, the blade should be cleaned or changed between samples. A stepwise manual for sampling soft tissue, bone, and teeth using only readily available, inexpensive instruments is provided in de Boer et al. [3]. Preferably, samples should be large enough to allow for re-sampling if the first attempt to obtain a DNA profile fails. Fragments that are submitted in total are best recorded in such a way that repatriation of the remaining portion after identification is still an option or to indicate that the remnant extract may need to be repatriated in lieu of any remaining sample.

### Reconciliation, identification, and quality control

As outlined in the INTERPOL DVI Guide, standards for identification should be based on reconciliation of antemortem and postmortem data from at least one primary identifier. In the case of fragmented remains, most matches will be based on DNA, and only a minority of fragments will provide another (corroborating) primary identifier such as odontology or fingerprints. While most jurisdictions will accept a single primary identifier, it is good practise to corroborate such an identification with available secondary identifiers such as associated material items (such as clothing, tattoos, or context from the scene). Alternatively, if resources are limited, the DNA sample from a case identified by another primary modality can be stored frozen without being processed. If questions later arise about that specific sample or identification, the DNA sample will still be available to test. Furthermore, the DNA from one fragment identified using dental methods can be used as a direct exemplar to re-associate additional fragments, instead of seeking sufficient familial DNA to make indirect matches; this avoids delays in processing family reference samples and simplifies matching statistics.

As an additional quality check, fragments identified using DNA should be compared with previously identified pieces from that particular individual. The best practice is to use a body diagram to illustrate which body parts were recovered and which are still absent [44]. Using such a diagram reveals DNA contamination by identifying duplicate body parts. The production of the diagram requires a detailed description of each fragment, preferably with photographs. This can be completed with the case file and augmented by CT images and information from the scene. Use of a body diagram can also assist in explaining levels of disruption to the next of kin (see below), which in turn can inform family decisions regarding viewing the remains. Any proposed identification should be thoroughly checked before proceeding to the identification board for formal identification and repatriation.

### Communication with families and judiciary

Incidents with many fragments pose specific challenges when communicating with the families of the deceased. Although best practice dictates separating and identifying all pieces originating from a single individual, small commingled fragments or segments of decomposed soft tissue may remain unidentifiable at the end of the process. The families should be made aware of this for transparency and local authorities should plan how to handle this issue.

Additionally, given the time-consuming process of recovering and identifying fragmented remains, it is likely that fragments from the same individual will be identified weeks, months, or even years apart. Consequently, families may have specific wishes as to how they should be kept informed. Implementing a process for next of kin to make these considerations early, with the freedom to change their minds at any time, can be facilitated by a form outlining several options. For example, some may want to be notified each time a new fragment is identified, while others may wish to be informed of only the first fragment or only at the end of the process. Experience shows that this is an individual decision and often changes as time from the incident extends. Furthermore, family members may express disparate views and wishes [45].

Consideration should be given to how the extent of disruption and the completeness of the body would be communicated to family members. At times, family members may request various types of information and differing levels of detail. To facilitate communication, the DVI team can present various prearranged options, ranging from full file review, to a review without photos, to more removed versions of the data such as diagrams. Following the 2015 Shoreham air crash, discussions were held between the forensic anthropologist, the senior coroner, and the family liaison coordinator to consider various options, including virtual three-dimensional reconstruction of the body parts using postmortem CT data. This idea was ultimately rejected as it was considered too graphic. Instead, a body diagram was used to convey each individual’s completeness. A table that listed the identified body parts was also created, with one column using scientific terminology and the other non-scientific terms.

Incidents in which a perpetrator’s remains are commingled with the victim’s remains pose unique challenges, as victims’ families often request that the perpetrator’s remains be separated from the victim’s. Depending on the condition of the remains and whether DNA or other forms of antemortem data are available for the perpetrators, this may or may not be possible. Communication on this should be clear and honest, to manage family expectations without promising unattainable goals.

Another important consideration is the presentation of evidence in courts and at coroners’ inquests. The way in which evidence is presented has been shown to impact how juries and families understand the evidence [46,47]. Discussions between the forensic anthropologist, police, imaging specialists, senior identification managers, judges, and coroners should therefore be held well in advance to determine how sensitive information will be represented. Sanitised images may be preferred. For example, at an inquest into the deaths of five children and their father at a remote farmhouse in Wales, line drawings were used to depict the location and condition of the remains within the house. These were created using Geographic Information System (GIS) survey data and geo-rectified photographs taken at the scene.

## Concluding remarks

The format of each DVI operation is dictated by the context of the event, including incident type, the number of victims, the condition of the remains, and the decisions made by local authorities, which typically include government officials such as emergency planners and councillors, coroners/public prosecutors, police, and other emergency services. As such, each DVI operation is unique, making it difficult to provide specific recommendations beyond the general ones presented in the INTERPOL DVI Guide. However, the combined experience of the authors illustrates similar specific challenges arising from disasters characterised by many fragmentary and compromised remains.

Communication between local authorities and forensic specialists is pivotal to ensuring a timely and efficient identification effort. Pre-disaster planning is essential and discussions between local authorities and forensic specialists should occur as soon as possible following the disaster. Agreements should be reached concerning the role of relevant forensic specialists at the scene and in the mortuary, and a flexible plan should be formulated for quality assurance, recovery, examination, and repatriation of remains.
